# Supplementary material for: Cloud BioLinux: pre-configured and on-demand bioinformatics computing for the genomics community
Source: BMC Bioinformatics. 2012 Mar 19;13:42. doi: 10.1186/1471-2105-13-42 (PMC3372431; doi:10.1186/1471-2105-13-42)
Supplement: Additional file 1 — Supplementary 1 Cloud BioLinux software documentation in the form of a mini, self-contained website. Users need to download and uncompress the .zip file, and open through a web browser the "index.html" file available on the main directory. (ZIP 1823 kb). [file 1471-2105-13-42-S1.ZIP › Cloud-BioLinux-Package-Documentation/docs/phylip.html]

Bio-Linux Software Documentation Pages

Back to search form

## phylip

|  |  |
| --- | --- |
| Name | phylip |
| Description | **Phylip is a suite of programs for inferring phylogenies.Methods in the package include parsimony, distance matrix, and likelihood methods including bootstrapping and consensus trees.**  The bio-linux-phylip package is a wrapper for the standard Debian/Ubuntu package and delivers, through its dependency on the phylip package, the phylip software and documentation. It also provides links to the documentation from the standard location in Bio-Linux systems, and some sampledata for phylip.  The key difference when you install the bio-linux-phylip package is that the phylip programs can be run by just typing their names on the command line. This is the usage expected on many systems, and written into much documentation. So, for example, on Bio-Linux, and many other systems, you would run the dnadist program by typing:  `dnadist`  on the command line. If you install only the Debian/Ubuntu phylip package, you would need to type:  `phylip dnadist`  **Note:** On Bio-Linux, there are two exceptions to the above. These are the phylip programs mix and factor. These program names are common enough to be used by other packages. Thus, to run these on Bio-Linux, you can either type:  `mix_phylip  factor_phylip`  or, since the Debian/Ubuntu package is also on your system, you can choose to run them by typing:  `phylip mix  phylip factor`  The programs distributed within the phylip package are:  - clique - consense - contml - contrast - dnacomp - dnadist - dnainvar - dnaml - dnamlk - dnamove - dnapars - dnapenny - dollop - dolmove - dolpenny - drawgram - drawtree - factor - fitch - gendist - kitsch - mix - move - neighbor - pars - penny - proml - promlk - protdist - protpars - restdist - restml - retree - seqboot - treedist  **References:**  Felsenstein, J. 1993. PHYLIP (Phylogeny Inference Package) version 3.5c. Distributed by the author. Department of Genetics, University of Washington, Seattle.    Felsenstein, J. 1989. PHYLIP -- Phylogeny Inference Package (Version 3.2). Cladistics 5: 164-166. |
| Homepage | http://evolution.genetics.washington.edu/phylip.html |
| Remote Documentation |  |
